# Next-generation probiotics: the upcoming biotherapeutics

**DOI:** 10.1007/s11033-024-09398-5

**Published:** 2024-04-15

**Authors:** Omnia Momtaz Al-Fakhrany, Engy Elekhnawy

**Affiliations:** Pharmaceutical Microbiology Department, Faculty of Pharmacy, Tanta University, Tanta, 31527 Egypt

**Keywords:** *B. Fragilis*, *Akkermansia muciniphila*, *Faecalibacterium prausnitzii*, *Roseburia* spp., Immunomodulation, Anti-inflammatory.

## Abstract

Recent and continuing advances in gut microbiome research have pointed out the role of the gut microbiota as an unexplored source of potentially beneficial probiotic microbes. Along the lines of these advances, both public awareness and acceptance of probiotics are increasing. That’s why; academic and industrial research is dedicated to identifying and investigating new microbial strains for the development of next-generation probiotics (NGPs). At this time, there is a growing interest in NGPs as biotherapeutics that alter the gut microbiome and affect various diseases development. In this work, we have focused on some emergent and promising NGPs, specifically *Eubacterium hallii, Faecalibacterium prausnitzii, Roseburia spp., Akkermansia muciniphila*, and *Bacteroides fragilis*, as their presence in the gut can have an impact on the development of various diseases. Emerging studies point out the beneficial roles of these NGPs and open up novel promising therapeutic options. Interestingly, these NGPs were found to enhance gastrointestinal immunity, enhance immunotherapy efficacy in cancer patients, retain the intestinal barrier integrity, generate valuable metabolites, especially short-chain fatty acids, and decrease complications of chemotherapy and radiotherapy. Although many of these NGPs are considered promising for the prevention and treatment of several chronic diseases, research on humans is still lacking. Therefore, approval of these microbes from regulatory agencies is rare. Besides, some issues limit their wide use in the market, such as suitable methods for the culture and storage of these oxygen-sensitive microbes. The present review goes over the main points related to NGPs and gives a viewpoint on the key issues that still hinder their wide application. Furthermore, we have focused on the advancement in NGPs and human healthiness investigations by clarifying the limitations of traditional probiotic microorganisms, discussing the characteristics of emerging NGPs and defining their role in the management of certain ailments. Future research should emphasize the isolation, mechanisms of action of these probiotics, safety, and clinical efficacy in humans.

## Introduction

The human body is colonized by diverse symbiotic microorganisms inhabiting the gastrointestinal tract and gut microbiota. The gut microbiome comprises up to 100 trillion symbiotic microorganisms, approximately ten times the number of eukaryotic cells in humans [1]. The human gut microbiome represents an unexplored store of potentially advantageous microbes that substantially impact the human host during homeostasis and disease progression. The metabolic byproducts and the immunomodulatory properties of certain bacterial species made these microbes crucial for human health. The gut microbiome will generate many active metabolites such as short-chain fatty acids, vitamins, and some compounds like analgesics, anti-inflammatory, and antioxidants that are beneficial for human health. This is accompanied by potentially health-damaging products such as neurotoxins, carcinogens, and immunotoxins [2]. They can affect the metabolic and immunologic processes when they enter the blood. Moreover, changes in intestinal microbiota composition could result in complex diseases such as neurodegenerative diseases, obesity, asthma, diabetes mellitus, inflammatory bowel disease, and others. Hence, a healthy gut microbiota is crucial for health promotion [3].

Scientific investigations have focused on studying pathogenic microbes and discovering suitable methods to prevent and treat subsequent diseases. On the contrary, certain bacteria species may benefit their host through a symbiotic relationship. In general, these microbes are called probiotic microorganisms. The FAO/WHO junction has defined probiotics as living microbes that, when utilized in appropriate quantities, confer positive health benefits to their consumers [4]. Most probiotic bacteria are Gram-positive and act through modulation and preservation of intestinal tract healthiness, such as *Lactobacillus* and *Bifidobacterium* [1].

Recently, most licensed microorganisms sold as probiotics belong to the lactic acid bacteria (LAB), typically represented by the genus Lactobacilli. They are metabolically characterized by the production of lactic acid from carbohydrates, causing an acidic environment that inhibits some pathogenic bacteria’s growth. Also, they may yield secondary metabolites, like exopolysaccharides, bacteriocins, and enzymes, that are valuable to human health [5]. Even with the benefits as mentioned earlier, recent probiotic trends tend to reduce the use of Lactobacillus probiotic groups and augment the usage of other bacteria that are more suitable to the intestinal environs [6]. These bacteria are designated as Next Generation probiotics (NGPs). In recent times, NGPs have gained increasing popularity owing to their great benefits compared to conventional probiotics. Profound research in this new-fangled generation of probiotics will pave the way for further targeted therapeutics to aid in managing emerging diseases [7].

Originally, probiotics regulation and marketing were developed in the food industry sector. Then again, these innovative strategies will influence the pharmaceutical and health sectors. This requires investigations in regulatory frameworks. That’s why; academic and industrial inquiries are now dedicated to identifying new microbial strains of gut origin for developing next-generation probiotics [8]. Currently, investigations on using probiotics and NGPs for the treatment and/or prevention of various human ailments have gained impetus. Besides, there is more interest in the biology of probiotics via sequencing genomes of probiotic microbes. Also, investigating the interactions of probiotic microorganisms with human cells and pathogenic microbes [9]. In-depth investigations into this novel probiotic candidate will pave the way for developing further targeted tools that help treat various disorders. The goal of this review is to provide an outline of some of the promising next-generation probiotics (NGPs), describe their characteristics, and point out their prospective health benefits and the future directions in this field.

## Probiotics: new species and health targets’ perspectives

### Probiotics and next-generation probiotics

The word “probiotics” originated from a Greek word meaning “for Life” [10]. The Food and Agricultural Organization (FAO) demarcates probiotics as “living non-pathogenic microbes, when consumed in appropriate quantities, give valuable health impacts to their host” [11]. Traditionally, fermented food was consumed for its nutritive and health-promoting therapeutic prosperities even before the identification of probiotics [12].

Conventionally, lactic acid-producing bacteria (LAB) such as *Lactobacilli*, *bifidobacteria*, and others have been used as probiotics. These microorganisms were principally isolated from fermented dairy products and fecal microbiota. By expanding our awareness of the gut microbiota and its roles, the future will hold an assortment of novel prospective approaches and novel probiotic taxa. However, most microorganisms inhabiting the human gut have remained unidentified because they are mostly anaerobic bacteria that are difficult to cultivate. Recently, with modern microbiology, particularly the polymerase chain reaction (PCR) of the 16 S rRNA gene, the next-generation sequencing (NGS) and bioinformatics, we have precisely identified and detected several bacterial strains residing in the gut. Thus, recent advances in complete genome sequencing and culture techniques have facilitated the isolation and characterization of various novel microbes from the human microbiome having promising health benefits and so probiotic potential. Therefore, they could be developed as next-generation probiotics (NGPs). They could be defined as “living microbes identified on the base of comparative microbiome investigations which confer health advantages to their host when taken to suitable extents.” Thus, the term ‘probiotic’ has been extended to hold more probiotic species next to the well-recognized lactic acid bacteria and bifidobacteria. These species are generally referred to as NGPs that have promising effects in managing inflammatory diseases, cancer, and metabolic disorders. NGPs belong to genera with no history of use as probiotics and are likely to be delivered under drug regulatory frameworks [13]. Examples include; *Roseburia intestinalis*, *Eubacterium* spp., *Akkermansia muciniphila*, *Faecalibacterium prausnitzii*, and Bacteroides spp [5].

In contrast to the traditional probiotics, NGPs have physiological utilities, e.g., the production of folate, serotonin, indoles, and short-chain fatty acids (SCFAs); butyrate, propionate, acetate and others), all of which have a major role in the regulation of physiological host phenotype. Transforming these novel species into industrially beneficial probiotics is a great challenge. This could be attributed to their need for expensive and complex rich culture media and anaerobic conditions. Therefore, research in this aspect for defining the optimal fermentation and industrial processes is a challenge [13].

### Sources of isolation of probiotics and NGPs

Isolation of potential probiotic strains is the principal step included in FAO/WHO guidance. Researchers come to an understanding that we still need novel strains of probiotic bacteria that could be used for specific treatments [14].

Traditional probiotics include bacteria of the genus of Lactobacillus and Bifidobacterium. It was claimed that probiotics intended for human use should be of “human or food origin.” This is attributed to the fact that those strains are expected to be safer for humans and their ability to bind to human intestinal epithelial cells [15].

**Figure. 1:**
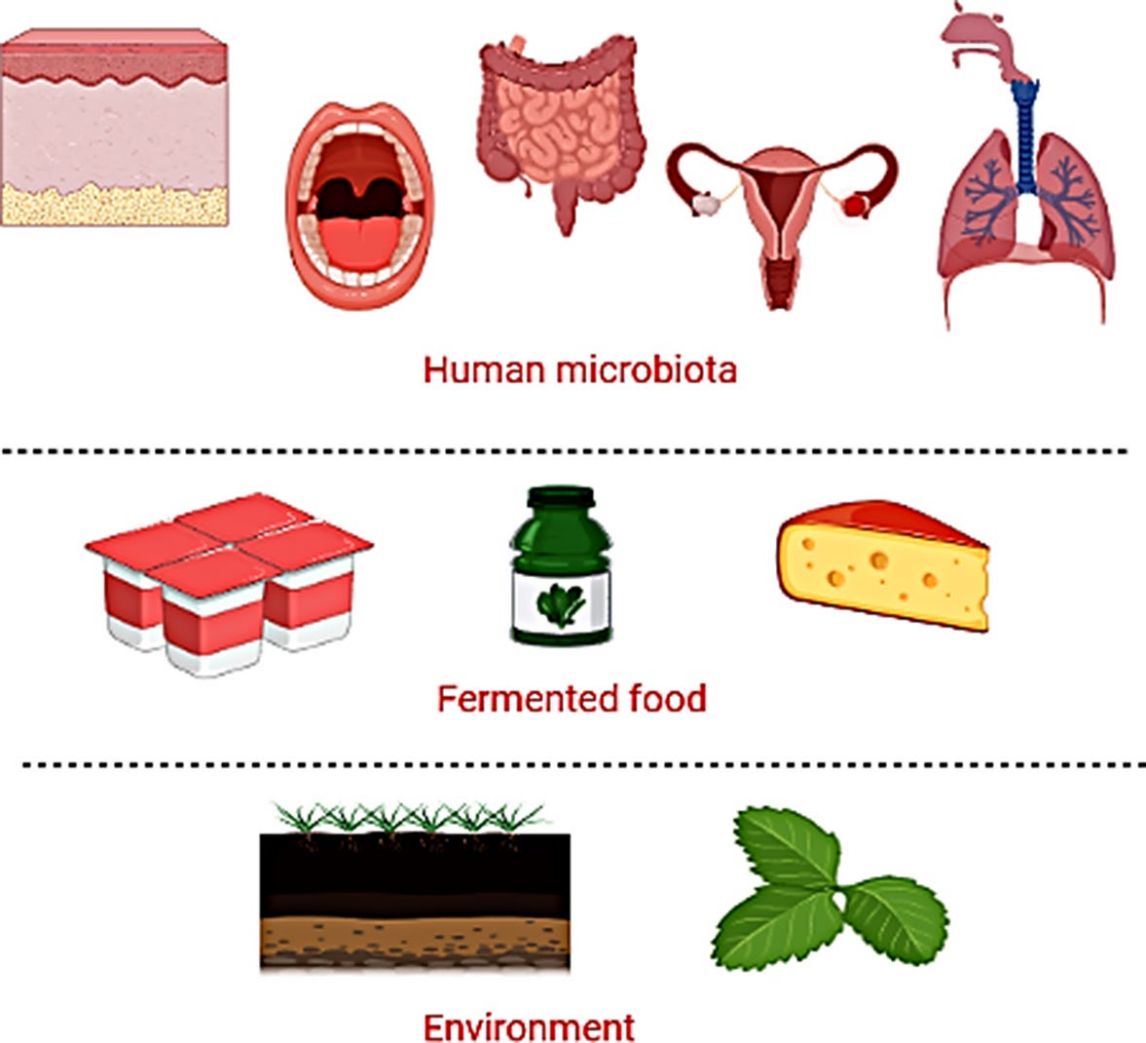
Sources of probiotics

The conventional sources of probiotic strain isolation are the gastrointestinal tract and breast milk. Human milk is an important factor in the colonization of the newborn intestine with the first microbiota. Thus, human milk is thought to be a source of bacterial strains with potential probiotic activity. Furthermore, the feces of infants, children, and adults have been found to be the best source of probiotics for human use. This is due to their ability to resist gastrointestinal transit and colonize the intestines for advantageous actions [16]. Besides, animal-origin food sources (milk, fish, meat products, and honey), along with fermented and non-fermented plant-origin food sources (examples include grains/cereals, fruits, dairy, and vegetables), could be additional sources for the isolation of probiotic strains [13]. This could be due to the thought that many well-studied probiotic strains isolated from human feces are not of human origin (e.g., *Bifdobacterium animalis* subsp. Lactis and *Saccharomyces cerevisiae* var. boulardii). Consequently, it is proposed that food is the primary source of microorganisms present in the gastrointestinal tract (Fig. 1).

Remarkably, fermented food is the main natural source of traditional probiotic strains belonging to LAB. Regular fermented food consumption has been linked to momentous health benefits, e.g. decreased risk of type II diabetes, cardiovascular diseases and a recognized advantageous metabolomics profile. Consequently, fermented food is expected to be the most important source of LAB in human gut microbiota and also has the prospective for future probiotic development approaches [17].

In addition to food, probiotics have been mainly isolated from the gut microbiome. However, the gut microbiome will not be the only basis for the isolation of novel probiotics. Niches for the detection of new species include the oral cavity, female urogenital tract, skin, and nasopharyngeal tract, in addition to environmental sources such as soil [13]. NGPs have been isolated with new techniques that allow isolation, identification, and modification of commensal bacterial species [18].

The innovative concept of probiotic use is “personalized” or “precision” treatment, which has the specific characteristics of the host, thus evading the “one size fits all” approach, which has proven unsuccessful. A personalized probiotic treatment strategy was recently suggested and verified in animal model studies for intestinal syndromes. Commensal bacteria isolated from the healthy host gut microbiota were stored in a ‘microbiota biobank,’ and after testing certain characteristics, the bacteria were effectively used as a therapy for dysbiosis-related diseases. This strategy assumes that commensal bacteria isolated in a personalized way could facilitate the colonization process based on the specific genetic makeup of their host [19].

#### Approaches for isolation of the NGPs

Actually, there are significant differences in the ways NGPs are studied compared to traditional probiotics. Detection of traditional probiotics is usually ascribed to a top-down screening approach. This includes screening of microorganisms in healthy persons compared to those in unhealthy ill persons. The “experience-first” technique directs the discovery of probiotic microbes. Yet, due to the lack of data on their principal ways, this technique must depend on continual investigations and complex screening procedures to determine their potential health benefits [20, 21]. Indeed, NGPs’ screening strategies are similar to drug development techniques that depend on two bottom-up development methods: phenotypic-based and target-based. The phenotypic-based approach involves screening based on the impacts of cells and animals on certain strains via in-vitro cultures, cells, and animal models of certain ailments.

On the other hand, the target-based approach greatly relies on sequencing techniques to assess and explore the capability of the bacteria or their metabolic products to form molecular effector molecules that can modulate host and/or microbial-related signaling pathways. Such studies will aid in determining the functions, safety, and possible molecular mechanisms of NGPs in animal models. Yet, some bacteria cannot be cultivated and isolated due to difficulties attaining the required anaerobic environment. Though novel culture media and modified techniques are in progress, including various culture conditions, rapid bacterial identification, and enhancing the growth of cultured bacteria and their potential as NGPs or biotherapeutics. The US FDA has launched a program called “Live Biotherapeutic Products” (LBP). LBP aims to regulate the clinical trials and commercial application of the evolving probiotic microbes. It is anticipated that NGPs will be developed as a significant tool in the health industry shortly [22].

### Characteristics and features of probiotics and NGPs

**Figure. 2:**
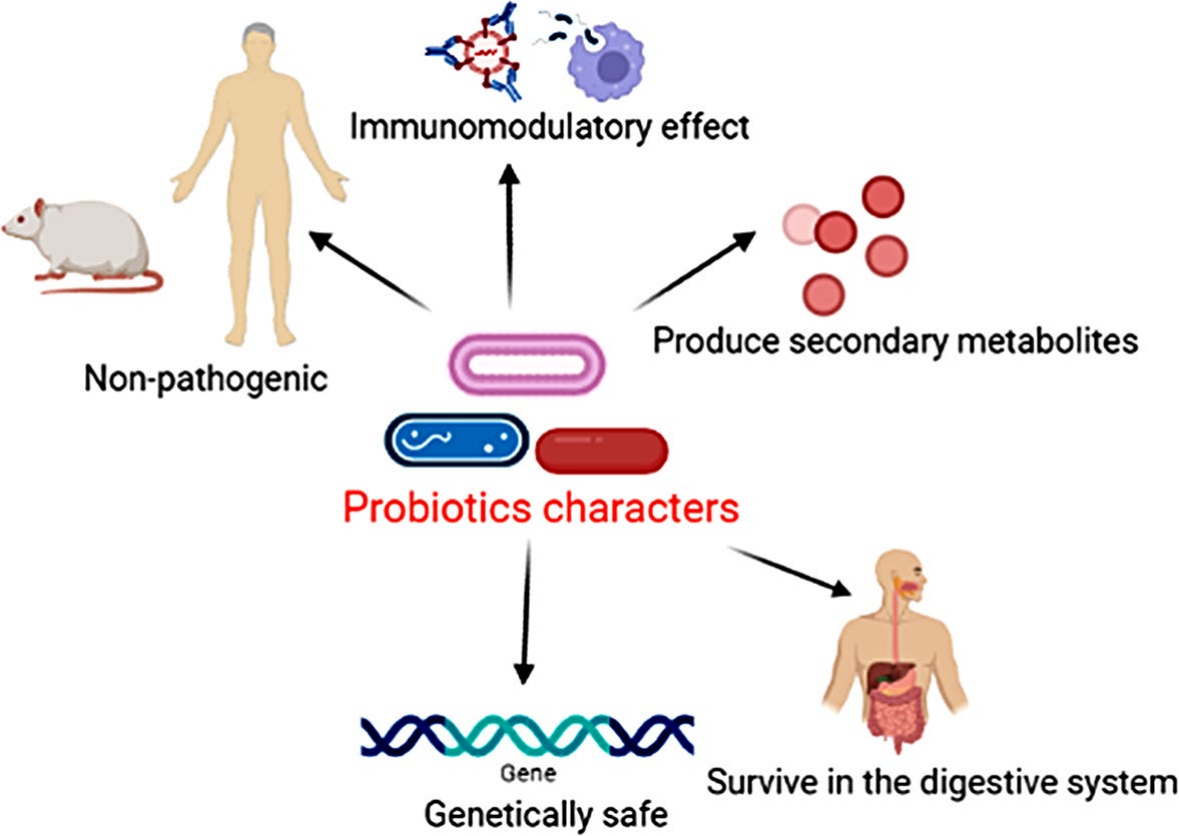
Characteristics of effective probiotic microorganism

Several researchers have documented the properties which a microorganism should have to be considered a probiotic. These features are summarized in Fig. 2.

Accordingly, probiotic microorganisms should have the characteristics that allow them to bear adverse conditions in their host. They can colonize their host and improve their healthy state by regulating their microbiome and carrying out biological roles [23]. As a general rule, the selection and characterization of novel probiotic strains should adopt basic selection criteria. In 2002, the United Nations Food and Agriculture Organization/World Health Organization (FAO/WHO) published guidelines that propose the diverse criteria which must be assessed for selection of probiotic microorganisms, viz.: antimicrobial activity, ability to adhere to epithelial tissues, resistance to harsh conditions inside the human, and safety for usage [23]. The safety of probiotics is typically well-defined by their source and absence of a link with any pathogenic cultures, along with their antibiotic resistance profile [24].

## Next-generation probiotics (NGPs)

Traditional probiotic microbes such as *Bifidobacterium* spp. and *Lactobacillus* spp. are generally recognized as safe and are effectively used in many diseases. Conversely, they are not disease-specific, and their efficacy on disease progression is still undefined. Accordingly, the identification and characterization of more potent and disease-specific NGPs represent a crucial research area for the scientific community worldwide. Many probiotic bacterial strains have been identified from the intestinal microbiome using novel next-generation sequencing techniques, and these NGPs have become possible sources of innovative therapeutics for various diseases [25]. According to the recently available information, NGPs include *E. hallii, Akkermansia muciniphila, Christensenella minuta, Prevotella copri, Faecalibacterium prausnitzii, Bacteroides thetaiotaomicron, Parabacteroides goldsteinii*, *Bacteroides fragilis*, Roseburia spp. and others [26].

### Health benefits of new generation probiotics

Humans have used probiotics as fermented food for a long time. However, the beneficial properties of these microbes were not well considered till recently [13]. The growing research in the field of gut microbiota has increased interest in using probiotics in different applications. The use of probiotics in the medical field has been almost confined to the Lactobacilli strains as being the most promising. Probiotics have been shown to decrease the access of pathogens to intestinal epithelial cells.

**Figure. 3:**
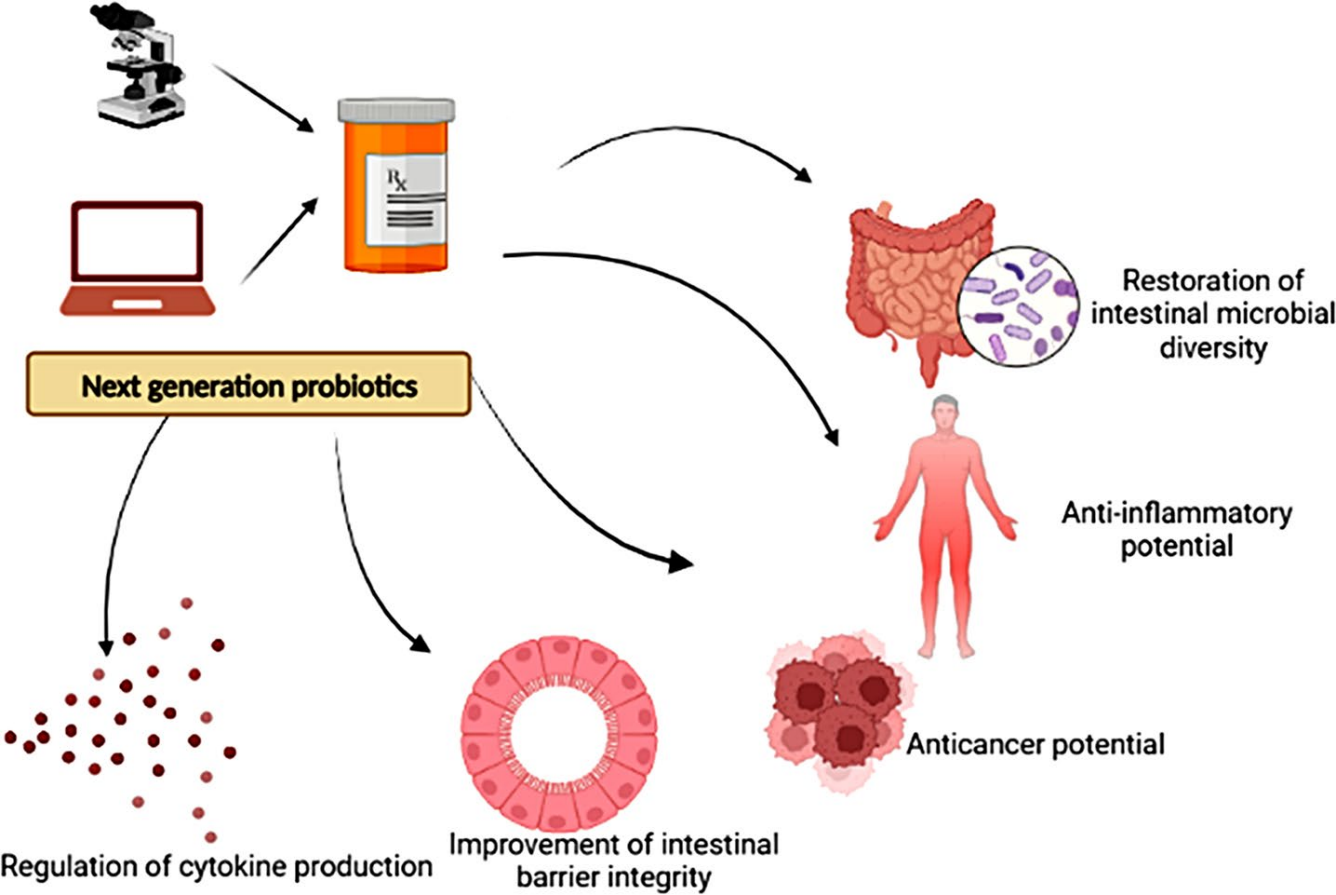
The possible therapeutic actions of the next generation probiotics

New-generation probiotics have a number of benefits over regular probiotics. Recently, NGPs were found to produce numerous beneficial metabolites (as indoles, secondary bile acids, folate, short-chain fatty acids (SCFAs); butyrate, propionate, acetate,) serotonin, gamma-aminobutyric acid (GABA), and others. These metabolites have a significant role in the regulation of the physiological host phenotype [27, 28]. In addition to the main cores of gut and immune homeostasis, various applications for probiotic therapy have emerged. These include liver disease, mood disorders, hypercholesterolemia subfertility, asthma, metabolic disorders and obesity [29–31]. The proposed actions of the NGPs as biotherapeutics are summarized in Fig. 3.

### Next-generation probiotics candidates

#### Faecalibacterium prausnitzii

*Faecalibacterium prausnitzii* is a highly promising next-generation probiotic. *F. prausnitzii* is considered one of the utmost abundant bacteria in the human intestinal tract. Taxonomically, *F. prausnitzii* belongs to family *Ruminococcaceae* family, phylum *Firmicutes* and class *Clostridia* [32]. Recent studies demonstrated a large genomic diversity among *F. prausnitzii* strains and found eight groups distinctive at the species level. Three novel species named *Faecalibacterium duncaniae, F. hattorii*, and *F. longum* were found to be originated from the human gut as determined using average nucleotide identity (ANI) analysis [23]. Also, *F. prausnitzii* strains can be categorized into two major phylogroups (phylogroups I and II), which were further assembled into five subgroups (I-A, II-B, II-C, II-D, and II-E) based on 16 S rRNA gene analysis [33]. It inhabits the gut of healthy individuals and represents about 5% of the total fecal microbiome [34]. Actually, a decline of *F. prausnitzii* in the gastrointestinal tract has been allied with microbial dysbiosis along with a variety of metabolic disorders and chronic immune-mediated ailments, such as inflammatory diseases and obesity [35, 36]. *F. prausnitzii* is usually utilized as an ideal biomarker for the identification of young obese persons with ulcerative colitis [37]. This could be attributed to its ability to encourage the synthesis of mucin and tight junction proteins and help restore damaged intestinal mucosa [38]. Furthermore, *F. prausnitzii* can control mucus secretion, differentiation of intestinal goblet cells, and glycosylation and retain the mucus barrier integrity as well. Based on the above-mentioned properties, *F. prausnitzii* is considered a probiotic that a key protective effect on the human intestine and its depletion will cause debilitated intestinal anti-inflammatory and immunomodulatory abilities [38].

Metabolically, *F. prausnitzii* is an anaerobic, non-spore-forming and non-motile, Gram-positive bacilli [39]. It is susceptible to oxygen and can hardly survive even in anaerobic culture conditions. However, adding cysteine, glutathione or riboflavin to the growth medium can augment the survival rate in a microaerobic culture [40]. A range of monosaccharides are utilized by bacteria as a energy source through the fermentation process. *F. prausnitzii* can ferment starch, oligofructose, fructose, glucose and inulin. Acetic acid can encourage its growth and yield carbon dioxide but not hydrogen. The most important products from glucose fermentation by *F. prausnitzii* are short-chain fatty acids such as formic acid, butyrate, a trivial amount of D-lactic acid, medium-chain fatty acids, and salicylic acid. The features have been associated with *F. prausnitzii’s* health benefits that involve immunomodulatory responses, better intestinal barrier integrity, and anti-inflammatory properties. This helps to maintain gut homeostasis [39]. *F. prausnitzii* has been reliably designated as one of the most significant butyrate producers in the gut [38]. The intestinal microbiota produces butyrate and has a key role in intestinal and body functions. Actually, butyrate is a main energy source for the intestinal epithelial cells, adjusts the activity of intestinal T-cells, inhibits invasion of pathogens, promotes apoptosis of colon cancer cells, prevents intestinal inflammation, regulates the immune system and helps to recover metabolic syndrome [40]. It produces useful effects through diverse pathways, such as the regulation of histone acetylation and mitogen-activated protein kinases. As an illustrative example of several pathways for electron disposal in the intestinal microbiota, *F. prausnitzii* can produce butyrate, and its simultaneous formation of NAD^+^ and reduced ferredoxin is able to modulate immune responses [38].

Salicylic acid Also, like butyrate, salicylic acid can block the NF-*κβ* activation and prevent the production of IL-8. Besides, salicylic acid can act as the forerunner of 5-aminosalicylic acid, a drug generally prescribed to manage Furthermore, *F. prausnitzii* produces peptides derived from the microbial anti-inflammatory molecule (MAM), inhibiting the host’s activation of NF-κB pathways The advantageous properties of these active metabolites include anti-inflammatory activities, preservation of gut barrier functions, gut immune homeostasis, and induction of apoptosis in colorectal cancer cells [40].

Considering the properties mentioned above, candidates of the genus *Faecalibacterium* have been investigated as modulators in cancer immunotherapy [44]. Researchers have found a relationship between the concentration of *Faecalibacterium* in the gut and the enduring survival of cancer patients. What’s more, scientists disclose that the number of regulatory T cells and the blood levels of the pro-inflammatory cytokines IL-2, IL-8, and IL-6 during melanoma metastases were adversely correlated with the quantity of *Faecalibacterium* in the intestine (Chaput et al., 2017). For these reasons, *F. prausnitzii* is considered a key therapeutic target and prognostic marker in melanoma patients (Gopalakrishnan et al., 2018).

At this point, *F. prausnitzii* isolated from healthy people has proven worthy of in-vitro anti-inflammatory and immunomodulatory activities. Even though its safety has been confirmed in a study on calves, its prospective role and safety in human beings are still indistinct [34].

#### Akkermansia muciniphila

*Akkermansia muciniphila*, a symbiont microbe colonizing the human intestinal mucosal layer, is a promising member of next-generation probiotics. *A. muciniphila* plays an imperative role in enhancing host metabolic functions and immune reactions. Besides, *A. muciniphila* could be significant in amending cancer therapy [34].

*A. muciniphila* is a non-motile, elliptic Gram-negative bacterium that forms no endospores. Taxonomically, it belongs to the family *Akkermansiaceae* and phylum Verrucomicrobia phylum. *A. muciniphila* commonly inhabits the intestine of humans throughout their first year of life to reach comparable levels in healthy adults. It is commonly distributed in the guts of animals and humans. The abundance of *A. muciniphila* in healthy people is more than 1 -5% [33]. On the other hand, obese patients with type II diabetes, hypertension, and liver disorders have a lower fecal abundance of *A. muciniphila*.

Metabolically, *A. muciniphila* can use the mucin-derivative monosaccharides: galactose, fructose and N-acetylglucosamine. These mucin-derivative constituents might be necessary supplements for the optimum growth of *A. muciniphila*. *A. muciniphila* was reported to be able to grow on synthetic culture media, in which a blend of N-acetylglucosamine, threonine glucose, and peptone substitutes mucin. This synthetic culture medium is suitable for cultivating *A. muciniphila* with very similar efficiency to the mucin medium, despite the fact that synthetic medium is free from any compounds which could be incompatible with human beings. Furthermore, the safety of *A. muciniphila* grown on a synthetic medium for human administration was established [33].

*A. muciniphila* is characterized by its ability to utilize intestinal mucins and glycoproteins from the epithelial mucus layer as its sole source of nitrogen and carbon. The intestinal mucosa primarily protects epithelial cells against infectious microbes and provides energy for the growth of the microorganisms that can utilize for nutrition. The capability of *A. muciniphila* to stick to intestinal mucosa is an advantageous probiotic trait. Low gut levels of *A. muciniphila* may end in thinning the mucosal layer. As a consequence, it is leading to the waning of the intestinal barrier homeostasis and facilitating toxins invasion of the host. The link between *A. muciniphila* and its host is not only revealed in the utilization of energy associated with glucose, lipid and protein metabolic rate but also in the mucosal layer integrity and the associated mucosal immune reactions. *A. muciniphila* contributes to the host immune system regulation and also improves the intestinal epithelial cells’ integrity and the mucosal layer thickness. In that way, *A. muciniphila* supports the intestinal healthiness [30].

Recently, *A. muciniphila* has been recognized to have a substantial role in human homeostatic and pathological conditions. Several in vivo and clinical research have investigated the link between the abundance of *A. muciniphila* and several ailments. Declined levels of *A. muciniphila* may be linked with the development of various diseases. These could include metabolic disorders and inflammatory ailments, such as obesity, inflammatory bowel disease (IBD), autism, and type 2 diabetes. *A. muciniphila* defends against obesity and type 2 diabetes by modulating the neuromodulatory system that controls glucose metabolism [39]. Plovier et al. have reported that treating *A. muciniphila* in pasteurized conditions at 70 °C for 30 min considerably raises the colon’s length and depth and enhances resistance to both obesity and insulin resistance. Patients with inflammatory bowel diseases and metabolic disorders were found to have lower levels of *A. muciniphila*, suggesting that this bacterium may have anti-inflammatory characteristics [40]. The capacity of *A. muciniphila* to aid the repair of the compromised intestinal barrier caused by a high-fat diet explains these positive benefits. However, Weir et al. reported that *A. muciniphila* levels were noticeably raised in colorectal cancer patients compared to healthy persons. This negative connection might be related to diet and medication. For instance, food intake was significantly diminished in patients with colorectal cancer, whereas fasting is could result in elevating the levels of *A. muciniphila*. A small sample size of patients may be another factor. Besides, *A. muciniphila* was found to enhance the anti-cytokine drugs used for the treatment of cancer in model animals [39].

Recent studies in the field of the microbiome have reported that beneficial therapeutic impacts are linked with probiotics in a viable form. Accordingly, *A. muciniphila* could represent a significant biomarker of the state of host healthiness by demonstrating disease progression. Surprisingly, Plovier et al. revealed that pasteurized *A. muciniphila* can also stop obesity with efficacy better than live bacteria. Moreover, the researchers reported the stability of the outer membrane protein of *A. muciniphila*, Amuc-1100, during pasteurization. They reported that Amuc-1100 could interact with Toll-like receptor 2 to enhance intestinal barrier function. Thus, Amuc-1100 could do part of the probiotic task by itself. In accordance with this outcome, Ottman et al. observed that Amuc-1100 may activate TLR2 and TLR4 to enhance IL-10 production. As a result, it could regulate the immune response and the intestinal barrier function. Hence, *A. muciniphila* is a promising target in microbiome-related ailments such as cancer, metabolic syndrome, colitis and immune syndromes [41].

#### Bacteroides fragilis

*B. fragilis* is a commensal, long rod anaerobic Gram-negative bacteria that belongs to the Bacteroides. *B. fragilis* could be transmitted from mother to child during delivery. Consequently, it becomes the main inhabitant of the human gut and represents about 1% of the gut microbiota. Still, its abundance in the female genital tract, mouth and upper respiratory tract is also evidenced. Bacteroides are opportunistic pathogens that are responsible for bacterial contagions, IBD and human tumor immunity [40]. Conversely, recent studies confirmed the probiotic properties of nontoxigenic *B. fragilis* strains. *B. fragilis* can stimulate the host adaptive immunity, inhibit inflammation, activate the maturation of the immune system, regulate the intestinal microbiome, and retain the gut health and homeostasis through Polysaccharide A (PsA) and other outer membrane vesicles of this NGP [41]. *B. fragilis* is useful for human health and have been suggested as a candidate probiotic. Previous studies evidenced that *B. fragilis* inhibits other pathogenic microbes by hindering their growth and/or translocation. *B. fragilis is* shown to inhibit *Clostrioides difficile* infection in animal models. Interestingly, treatment with this probiotic significantly enhanced the bacterial diversity and positively affected the abundance of *(A) muciniphila.* It was reported that *(B) fragilis* repressed *(C) difficile* adherence by inhibition of apoptosis, zonula occludens-1 (ZO-1) and (mucin-2) MUC- 2 loss. Accordingly, *B. fragilis* conserved the intestinal barrier functions [26, 32].

Other studies detected the competitive inhibitory activity of *B. fragilis* against the translocation of *Salmonella* Heidelberg [42]. This activity was explained by the production of the antimicrobial protein-1 (BSAP-1), which has membrane attack/perforin (MACPF) domains which lyse bacteria infecting the host cells [43]. Other factors may have a significant role in such competitive antagonism. These may include the eukaryotic-like ubiquitin protein (BfUbb) and contact-dependent Type VI secretion system (T6SSs) [44].

Latest studies revealed that *B. fragilis* metabolizes various carbohydrates in the colon and produces eight distinct capsular polysaccharides, of which polysaccharide A (PSA) is a distinctive amphoteric polysaccharide and an immune modulator [44]. PSA is internalized and processed by the antigen-presenting cells for recognition by T cells. A fact that suggests the continual boosting of host immunity with PSA [44]. In fact, PSA stimulates the differentiation of initial T cells into Treg cells by adjusting the dendritic cells. Also, PSA enhances the expression of Foxp3 and CD39, hinders the inflammatory cytokine IL-17, and induces the production of IL-10, which aids in the management of intestinal inflammatory disorders [45]. Therefore, treatment with either *B. fragilis* or the purified PSA is regarded as an innovative therapeutic strategy for human autoimmune syndromes and IBD. Recently, *B. fragilis* was reported to be useful in cancer patients by enhancing the immune system functions, reducing LPS-related signaling, improving the activity of gut microflora, and preventing leaky gut by preserving intestinal barrier homeostasis. Actually, it is predictable to be a viable therapeutic. A preclinical study used *B. fragilis* as a living biological therapy and reported its significant efficacy and safety. Thus, *B. fragilis* provides a novel therapeutic for ulcerative colitis (UC). SK08 live bacterial powder, a living biological drug prepared to utilize *B. fragilis*, has been accepted by the Chinese Food and Drug Administration. At present, SK08 live bacterial powder is in the clinical trial stage. Now, the drug is listed and categorized as a therapeutic biological drug.

#### Eubacterium hallii

*E. hallii* is another potential NGP for forthcoming probiotic biotherapeutic preparations. *E. hallii* is a Gram-positive, uniform or pleomorphic, non-spore-forming, and obligately anaerobic rod-shaped bacteria that belong to the phylum *Firmicutes*, genus *Eubacterium*. *Eubacterium* spp. Represent one of the main gut microbiota and show extensive colonization of the intestinal tract in several human populations and has an abundance of just about 3% in adults. *E. hallii* is a chemoorganotrophic bacterium that can utilize different carbon sources, including sugars and organic acids [46]. *E. hallii* produces two significant short-chain fatty acids (SCFAs), propionate and butyrate. SCFAs play fundamental roles in maintaining gut health, including; improving mucus production, stimulating proliferation and differentiation of the enterocytes, and enhancing the epithelial cells’ health. Depletion of SCFAs will prompt an inflammatory response [47]. Amongst the SCFAs, propionate, and butyrate are recognized to be beneficial to human health. *E. hallii* was first described as a butyrate producer in the human intestine by Barcenilla et al. [46]. Butyrate can be produced in the gut from carbohydrates through the process of glycolysis followed by stepwise transformation to finally produce butyrate. Pham et al. [47] have reported that *E. hallii* is a principal producer of butyric acid in the newborn gut.

Propionate can be produced by gut microbes through sugar fermentation. While *Eubacterium* spp. can ferment complex carbohydrates, definite strains of *Eubacterium* spp. may can’t ferment specific complex carbohydrates. These strains depend on the metabolic products of other gut microbes for doing so. Thus the fermented metabolites formed by the other gut microorganisms may then be used by *Eubacterium* spp. Several studies demonstrated the significance of these cross-feeding mechanisms in SCFA formation by *Eubacterium* spp [48]. . These investigations involved the co-culturing of *Eubacterium* spp. with *Bifidobacterium* spp. in culture media having complex carbohydrates. *Bifidobacterium* strains that are able to degrade complex carbohydrates, such as fucosyllactose and arabinoxylan oligosaccharides were reported to produce 1,2-propanediol, acetate, and lactate. Then all of these metabolites were taken up and utilized by *Eubacterium* spp. to yield butyrate and propionate. Such cross-feeding mechanisms adopted by *Eubacterium* spp. Clearly highpoints the synergistic interactions among gut microbes and butyrogenic properties of complex carbohydrates. Furthermore, these mechanisms give emphasis to the ecological roles of *Eubacterium* spp. in the gut ecosystem. In the early periods of life, *E. hallii* acts together with *Bifidobacterium* infantile to ferment the intermediate compounds of the breast milk oligosaccharides in order to produce SCFAs [48, 49]. The roles of *E. hallii* in obesity and diabetes were investigated by Udayappan et al. [50]. The authors revealed that *E. hallii* could metabolize butyric acid to activate the G-coupling protein receptor signaling pathway, improve GLP1 and GLP2 production, strengthen the intestinal barrier function, do not have an impact on the body weight and/or food consumption, and improve the insulin sensitivity and the energy metabolic rate. Thus it could be safe and effective in insulin sensitivity [51, 52].

Recent studies have evidenced that *Eubacterium* spp. carry out crucial metabolic transformations in the intestine with encouraging impacts on human health. Amongst these effects, the detoxification of toxic compounds into further benign forms appears to be valuable [53, 54]. In recent times, *E. hallii* has been reported to perform various useful transformations. For instance, Fekry et al. [55] observed the proficient transformation of the greatly abundant food-derived heterocyclic aromatic amine carcinogen – 2-amino-1-methyl-6-phenylimidazo (4,5-*b*) pyridine (PhIP) into a biologically unobtainable form 7-hydroxy-5-methyl-3-phenyl-6,7,8,9-tetrahydropyrido (3′,2′:4,5 imidazo (1,2-α) pyrimidine-5-mum chloride (PhIP-M1) by *E. hallii.* Moreover, PhIP is transformed by *E. hallii* in the presence of simulated distal and proximal colon microbiota resulting in a 120-fold and 300-fold rise in its abundance, correspondingly. This indicates its great perspective as a protective therapeutic. Also, in the same study, Fekry et al. [55] detected the antimicrobial activities of *E. hallii.* The authors showed that *E. hallii* is capable of breaking down glycerol into 3-hydroxypropionaldehyde (3-HPA), which exists in the form of reuterin in aqueous solutions. Interstingly, reuterin has been confirmed to have inhibitory activities against fungi, yeast and both Gram-negative and Gram-positive bacteria, probably by increasing the oxidative stress by modulating the intracellular glutathione. In that way, it could be an attractive biotherapeutic [56, 57].

Furthermore, *Eubacterium* spp. was found to contribute to gut and hepatic well-being by modulation of the bile acid metabolic profile. Recently, modulation of bile acid metabolism and/or gut microbiota are being investigated as innovative therapeutic strategies for hepatocellular carcinoma (HCC) and colorectal carcinoma (CRC) [56].

#### Roseburia spp

*Roseburia* spp. is a Gram-positive, obligate, anaerobic, curved rod-shaped bacteria. It belongs to the phylum Firmicutes, class Clostridia, order Clostridiales, and family Lachnospiraceae subgroup Clostridium XIVa. Its abundance in the gut of a healthy adult is about 3–15%. *Roseburia* species use complex polysaccharides and produce SCFAs (propionate, butyrate, acetate) [58, 59]. It is beneficial in inflammation, Parkinson’s disease, and inflammatory bowel disease (IBD) and is well thought-out as a candidate for NGPs. Remarkably, recent studies revealed the reduction of atherosclerosis in mice which were fed on both Roseburia and a diet rich in fibres. This was attributed to a high-fibre diet, which mediated butyrate formation by Roseburia to decrease atherosclerosis [60].

Moreover, recent studies reported the role of *Roseburia intestinalis*-derived flagellin, a significant structural constituent of the bacterial flagellum, in the cure of alcoholic fatty liver and ulcerative colitis. For instance, Seo et al. [61] observed that oral intake of *Roseburia intestinalis*-derived flagellin greatly restored the integrity of intestinal epithelial cells in the alcoholic fatty liver mice model and inhibited the potential intestinal fistula. Likewise, Wu et al. [62] have reported that *Roseburia intestinalis*-derived flagellin improves colitis. The authors detected its inhibitory activities on inflammatory bodies, which prompted apoptosis and intestinal inflammation in the colitis mice model. However, the clinical information on Roseburia is quite deficient. Further work is needed to justify the usage of Roseburia in various human ailments [31, 63].

#### Prevotella copri

*P. copri* is another NGP that belongs to phylum Bacteroidetes phylum. *P. copri* was reported to improve both glucose tolerance and liver glycogen levels [64]. It has been recognized as a potential target for metabolic disorders such as type-II diabetes and obesity [65].

#### Parabacteroides goldsteinii

*P. goldsteinii* is a next-generation probiotic that is highly recommended for obesity. Also, *P. goldsteinii* has displayed significant anti-inflammatory and insulin-stimulating properties [65].

#### Clostridium butyricum

*C. butyricum* is a spore-forming and obligate anaerobic Gram-positive bacteria. This microbe is named ‘butyricum’ because it ferment non-carbohydrates digestible and produce high levels of butyric acid. This is extremely significant for enterocyte proliferation and has a major role in maintaining colon health [7, 66]. In 2020, Chen et al. reported the anticancer activities of *C. butyricum.* The authors observed its ability to reduce the formation of intestinal tumors in mice. Furthermore, *C. butyricum* greatly decreased the proliferation of intestinal cancer cells and induced apoptosis. Interestingly, treatment with *C. butyricum* probiotic strains along with antidepressants caused a substantial improvement in depressed persons [67, 68].

## Future therapeutic prospects of NGPs

Traditional probiotics of the current generation have already proven their value in conserving the gut microbiota and decreasing inflammatory responses, allergic disorders, and autoimmune ailments [33, 69]. Nevertheless, next-generation probiotics have prospective beneficial features to human health, which extend the probiotics’ spectrum and participate in the development of innovative food products that meet the population’s increasing demands regarding health and quality of life. This makes NGPs an outstanding research area for both the pharmaceutical and the food industry.

Regardless of the restrictions, advances in developing NGPs are going on. So as to be used as biotherapeutics, NGPs must go through a series of clinical trials, including preclinical, toxicological, and pharmacodynamic studies [70, 71]. Consequently, future microbiome research must give emphasis to clinical translational investigations. At this time, there is deliberate progress concerning the therapeutic benefits of fecal microbiota transplantation (FMT) in recurrent C. difficile-associated infections. The emergence of NGPs has enhanced the investigations into human pathology and physiology. Targeted living biological preparations of NGPs could be a novel microbial remedy in preference to FMT for specific diseases [72]. In 2020, several intestinal microbiome drugs were announced to be in phase III clinical trial that can successfully decrease the threat of recurrence of C. difficile infections. Examples include SER-109, announced by Seres Therapeutics (2020) and RBX2660, announced by Rebiotix Inc (2020). Also, 4D Pharma (2020) stated the positive progress in phase I/II clinical trials from its phase II BHT-II-002 trial of Blautix®, which is a single strain live biotherapeutic to treat irritable bowel syndrome (IBS). Besides, in *ClinicalTrials.gov* system, accessed on 1 July 2023, there are registered trials concerning *A. muciniphila* (*ClinicalTrials.gov* identifier: NCT04797442, NCT05114018, NCT05720299 NCT02637115) and *F. prausnitzii* (e.g., NCT04938843, /NCT02908360, NCT02538354). These clinical trials have highlighted the efficacy of NGPs in the management of type II diabetes, other metabolic disorders, atopic dermatitis, asthma, allergic rhinitis and Crohn’s disease.

Despite the strong concern of the scientific community to extend the range of probiotic microbes, there are substantial obstacles for both the research and the industrial field. The most significant concerns are the safety and effectiveness of NGPs and the technological components of employing NGPs in food and therapeutic preparations. Most of these microbes have metabolic activities that make them challenging to use in large-scale food production [73, 74]. More in vivo studies and clinical trials are still needed to establish the effectiveness and safety of these microbes. Moreover, few studies have investigated the potential use of next-generation probiotics in food products and their influences on intrinsic technical and sensory factors. As a final point, emerging healthcare challenges, industry trends and consumer preferences will drive the mandate for incorporation of NGPs into a variety of formulations, supported by progressions in the delivery systems and the quality assurance to be implemented in healthcare policy and practice [75, 76], despite the fact that the human gut will remain the core of these therapeutics, clinically established applications will continue to develop in the immune system, nervous system, urogenital tract, cardio-metabolic system, respiratory system, skin, oral cavity, and the weight-management field. Sooner or later, these microorganisms may be employed as biotherapeutic preparations and nutritional supplements for managing various ailments.

### Global health challenges and regulatory issues

Probiotics and NGPs may play a significant role in facing emerging healthcare challenges, including drug-resistant infectious and metabolic diseases. The growing antimicrobial resistance is a global concern [77]. Certain NGPs strains have shown efficacy in decolonizing antimicrobial-resistant microbes from the human gut. Also, they have protected against multidrug-resistant infectious microbes thru their immunomodulatory effects on the microbiome and human immune system [27, 78]. This suggests their prospective roles in reducing the problems associated with antimicrobial resistance.

Additionally, NGPs and their antimicrobial metabolites are being explored as novel alternatives to antibiotics [13, 79]. In the past few years, coronaviruses, severe acute respiratory syndrome coronavirus (COVID-19) [80, 81], have formed massive healthcare, social, and economic challenges worldwide. Due to the rising mutation and time needed to develop effective vaccines, safe, low-cost, non-specific anti-inflammatory, immunomodulatory and antiviral agents were used as rescue treatments [30, 82]. Probiotics and NGPs have been proposed as part of preventative and acute care approaches for COVID-19 infection. This is due to their immunomodulatory activities and the effectiveness of certain probiotics in inhibiting upper respiratory tract viral contagions and minimizing the risk of ventilator-associated pneumonia. d’Ettorre et al. [83] reported reduced morbidity and mortality in a small group of hospitalized COVID-19 patients when standard therapy was supplemented with a multi-strain probiotic formulation. This indicates the potential of investigating probiotics and NGPs regarding their safety and effectiveness as adjuvant therapy [84, 85].

Even though certain NGPs are effective in a range of healthcare issues, their regular use in nutritional and healthcare applications remains restricted to some extent. Both the healthcare specialists and the consumers are still suspicious about their prospective usefulness and the level of clinical proof. This is greatly attributed to the regulatory environment. Probiotics hold different regulatory status among different countries and further differ in their considerations for application in various product formulations, health claims, labelling, and other user communication [12, 13]. While many countries have effectively employed specific regulatory frameworks and approved evidence-based probiotic claims, other countries’ approved claims are rare, and therefore, the communication about their health benefits in the market is limited [86, 87].

The European framework significantly impacts the future of industry-driven probiotic research due to its strict requirements and excessive market size. Therefore, the scientific and technical requirements for putting products, food and dietary supplements in the market and for claims greatly vary around the world. Investors working under diverse jurisdictions need to act in accordance with these different frameworks [88, 89]. A number of evidence-based categories and criteria might be taken into consideration. Further international synchronization should be encouraged. Convergent frameworks adapted to the forthcoming scientific discoveries will be a major application challenge for future probiotics and NGPs. Thus, a conciliation between scientific recognition and regulatory approval is the approach forward, and more harmonization of the regulatory frames will improve the implementation of probiotic products in the nutritive and healthcare industries.

### Safety profiles and implementation of NGPs

Most NGPs are anaerobic bacteria that are difficult to cultivate. In addition, other issues need to be solved. The exact dose of each NGP to produce its beneficial effects on humans must be determined. A recent study defined the dose of *A. muciniphila* as 10^10^ CFU. The particular dose of each other NGP must also be ascertained. Also, the optimal formulation for each NGP has to be determined to enhance bacterial survival for the duration of storage and in the harsh conditions of the gut microenvironment [85, 90]. Additionally, further studies on the concomitant administration of two or more NGPs to determine possible synergistic actions that may have beneficial additive effects. Still, additional research in this field is needed to avoid any consequent probiotic-related adverse effects [91, 92]. Although they are underestimated in human trials, the available reports show slight gastrointestinal disorders associated with consuming probiotics. More severe complications caused by invasive infections, such as bacteremia, sepsis or endocarditis, have been rarely detected and typically noted in immunocompromised patients [93, 94]. Other proposed concerns involve the lateral transfer of antibiotic resistance genes from the probiotic strains to gut bacteria and great immune stimulation, which may result in autoimmune diseases in susceptible individuals. Yet, none of these conditions have been reported. Consequently, safety regulations must be strictly executed and updated. For example, in the United States, regulations from the Food and Drug Administration (FDA), with the characterization of “generally recognized as safe” (GRAS), should be applied. Nevertheless, as novel NGPs are evolving, the regulations should be regularly updated, and the safety of NGPs should be coordinated with the needs and the safety of individuals [47, 85].

## Conclusions

Recent outlooks on the role of the microbiome in the maintenance of human health have sparked the expansion of next-generation probiotics that have positive impacts on human health through their microbiota modelling effect. NGPs are considered a striking development over traditional probiotics with a history of safe usage in humans. Recently, *Faecalibacterium prausnitzii*, *Akkermansia muciniphila*, *Bacteroides fragilis*, *Eubacterium hallii*, *Roseburia* spp., and others have emerged as NGPs for disease treatment. Also, specific strains have positive impacts on the human immune system and gastrointestinal and metabolic disorders. At the moment, various research institutions, pharmaceuticals, and food industries all over the world are dedicated to advancements in NGPs to verify their safety and effectiveness as biotherapeutics. The therapeutic effects of NGPs must be carefully well thought-out. As additional supplementary active microbes, the adverse reactions of these microbes, harmful effects of probiotic metabolites, gastrointestinal side effects, antibiotic resistance genes, skin reactions, and abnormal immune system stimulation must be carefully evaluated. Much remains to be understood about the metabolic activities, immunomodulatory effects, and ecological roles of next-generation health-promoting probiotic microorganisms. Thus far, based on evidence to date, there are many reasons for optimism. Future in-depth studies into the mechanisms of action of these NGPs will allow their use as biotherapeutics for the treatment of various disorders.

## Data Availability

All data are available in the manuscript.
